# *Staphylococcus aureus* from ocular and otolaryngology infections are frequently resistant to clinically important antibiotics and are associated with lineages of community and hospital origins

**DOI:** 10.1371/journal.pone.0208518

**Published:** 2018-12-06

**Authors:** Jenna I. Wurster, Paulo J. M. Bispo, Daria Van Tyne, James J. Cadorette, Rick Boody, Michael S. Gilmore

**Affiliations:** 1 Infectious Diseases Institute, Department of Ophthalmology, Harvard Medical School, Massachusetts Eye and Ear Infirmary, Boston, Massachusetts, United States of America; 2 Department of Microbiology and Immunobiology, Harvard Medical School, Boston Massachusetts, United States of America; 3 Henry Whittier Porter Bacteriology Laboratory, Massachusetts Eye and Ear Infirmary, Boston, Massachusetts, United States of America; Rockefeller University, UNITED STATES

## Abstract

*Staphylococcus aureus* is an important human pathogen that causes serious antibiotic-resistant infections. Its population structure is marked by the appearance and dissemination of successful lineages across different settings. To begin understanding the population structure of *S*. *aureus* causing ocular and otolaryngology infections, we characterized 262 isolates by antimicrobial sensitivity testing and multilocus sequence typing (MLST). Methicillin-resistant *S*. *aureus* were subjected to SCC*mec* typing and Panton-Valentine leukocidin (PVL) screening. Although we detected a high level of genetic diversity among methicillin-sensitive (MSSA) isolates, (63 sequence types—STs), the population was dominated by five lineages: ST30, ST5, ST8, ST15 and ST97. Resistance to penicillin, erythromycin and clindamycin was common among the major MSSA lineages, with fluctuations markedly impacted by genetic background. Isolates belonging to the predominant lineage, ST30, displayed high rates of resistance to penicillin (100%), erythromycin (71%), and clindamycin (63%). Overall, 21% of the isolates were methicillin-resistant (MRSA), with an apparent enrichment among otitis and orbital cellulitis isolates (>40%). MRSA isolates belonged to 14 STs grouped in 5 clonal complexes (CC), however, CC5 (56.1%) and CC8 (38.6%) dominated the population. Most CC5 strains were SCC*mec* type II, and resembled the hospital-adapted USA100 clone. CC8 strains were SCC*mec* type IV, and 86% were positive for the PVL toxin, common features of the community-acquired clone USA300. CC5 strains harboring a SCC*mec* type IV, typical for the USA800 clone, comprised 15.5% of the population. USA100 strains were highly resistant to clindamycin, erythromycin and levofloxacin (100%), while USA300 strains were frequently resistant to erythromycin (89%) but displayed lower rates of resistance to levofloxacin (39%) and clindamycin (17%). Our data demonstrate that the ocular and otolaryngology *S*. *aureus* populations are composed of strains that are commonly resistant to clinically relevant antibiotics, and are associated with the major epidemic clonal complexes of both community and hospital origins.

## Introduction

*Staphylococcus aureus* is a prevalent community-acquired pathogen as well as a leading cause of nosocomial infection [1, 2]. The global success of *S*. *aureus* is, in part, due to its ability to efficiently colonize the respiratory system and other epithelial and mucosal surfaces in healthy individuals, which serve as staging grounds for dissemination and infection [3, 4]. Additionally, the ability of *S*. *aureus* to develop resistance to multiple antibiotic classes promotes the selection and expansion of epidemic antibiotic-resistant lineages that can thrive in both the community and hospital settings, posing a serious threat to public health [5]. Community-acquired methicillin-resistant *S*. *aureus* (CA-MRSA), which cause predominantly skin and soft tissue infections, have rapidly spread throughout the United States following the first reported cases in the late 1990s [6]. MRSA have also become leading causes of clinically relevant infections of the eye [7, 8] and ear [9], which develop primarily in patients within the community setting and can result in drastic vision loss and poor patient outcomes [10].

Numerous comprehensive surveillance studies have performed genetic characterization of *S*. *aureus* isolates in the USA, however these have been primarily focused on systemic or skin and soft tissue infections [11–14]. Antimicrobial resistance surveillance studies of ocular and otolaryngology isolates have been performed previously on a local [15–18] or nationwide scale [7, 19], but molecular typing to characterize population structure is not a common component of those studies. To fill this gap, we sought to investigate the molecular epidemiology of *S*. *aureus* isolated from eye, ear, and sinus infections treated at a tertiary care center in New England. We used antimicrobial resistance testing and sequence typing to determine the population structure of the *S*. *aureus* reservoir in this setting, and to determine whether the major genetic lineages display varying rates of resistance to clinically relevant antibiotics.

## Methods

### Bacterial isolates

This study was approved by the Massachusetts Eye and Ear Institutional Review Board. In total, 262 *S*. *aureus* isolates were included in this study (Table 1). Sinus samples reflect swabs of sinus secretions identified on endoscopic examination, or sinus puncture in some cases. Purulent discharges from otitis cases were collected with a sterile swab and in some cases middle ear fluids were collected via tympanocentesis. Ocular samples were obtained via corneal scraping, conjunctival swabbing, or aqueous/vitreous aspirate. Suppurative collections from infected soft tissues of the orbit (orbital cellulitis) were collected with a sterile swab. Patient specimens were cultured on Blood Agar (sheep’s blood, 5%, Remel), Chocolate Agar (Remel), and MacConkey Agar (Remel) at 37°C. Suspected *S*. *aureus* colonies were routinely identified using a combination of phenotypic methods including detection of coagulase and protein A by latex agglutination, followed by confirmation of species and antimicrobial susceptibility testing using the MicroScan Walkaway 40 Plus System (Beckman Coulter, Brea, CA). Isolates were stored at -80°C in Microbank cryopreservative tubes (ProLab Diagnostics). Frozen isolates were cultured twice on blood agar before further molecular analysis.

**Table 1:** Distribution of infections from which *S*. *aureus* isolates were selected for antimicrobial susceptibility testing and molecular typing.

Body Site/Diagnosis	No (%) of cases		*P* value MSSA *vs* MRSA^2^
	*S*. *aureus* cases	Proportion of MRSA	
**Ocular Infections**	**56**	**15 (26.8)**	
Keratitis	25	5 (20.0)	1.00
Orbital Cellulitis	17	7 (41.1)	0.07
Conjunctivitis	13	3 (23.0)	1.00
Endophthalmitis	1	-	-
**Aural Infections**	**67**	**21 (32.3)**	
Otitis	28	12 (42.8)	0.01*
Not Specified^1^	39	9 (24.3)	-^3^
**Sinus Infections**	**139**	**21 (15.1)**	0.1
**Total**	**262**	**57 (21.7%)**	

^1^Patients who presented with ear inflammation and otorrhea. Based on the information sent to the clinical laboratory it was not possible to classify cases as otitis media or otitis externa.

^2^Statistical significance was calculated using the Fisher’s exact test performed on the GraphPrism.

*A *P* value <0.05 was considered significant.

^3^P values were calculated on a by-diagnosis basis. As such, non-specified aural infections were excluded from statistical analyses.

### Antimicrobial susceptibility testing

Antimicrobial susceptibility profiles were determined for all isolates by broth dilution using a MicroScan Walkaway 40 Plus System. Minimum inhibitory concentration breakpoints, as established by the Clinical Laboratory Standards Institute [20], were used to categorize the isolates as susceptible, intermediate, or resistant. Because we detected less than ten intermediately resistant isolates from all 262 tested *S*. *aureus*, intermediately resistant isolates were grouped with resistant isolates for determination of resistance rates. All isolates were tested against, clindamycin, daptomycin, erythromycin, gentamicin, levofloxacin, linezolid, oxacillin, penicillin, tetracycline, trimethoprim-sulfamethoxazole (TMP/SMX) and vancomycin.

### Multi-locus sequence typing

DNA purification from bacterial lysates was performed using Chelex 100 molecular biology resin (BioRad) as previously described [21]. Purified genomic DNA was diluted 1:10, and was assessed for purity and DNA concentration using a Synergy 2 Multi-Mode Plate Reader and Take3 software system (BioTek). PCR amplicons of seven *S*. *aureus* housekeeping genes (*arcC*, *aroE*, *glpF*, *gmk*, *pta*, *tpi*, and *yqiL*) were generated using the protocol indicated in the *Staphylococcus aureus* MLST database (http://saureus.beta.mlst.net) using Q5 Polymerase (New England BioLabs). Following visualization via gel electrophoresis, amplicons were subjected to Sanger sequencing by Genewiz Incorporated (South Plainfield, NJ). Raw sequencing traces were aligned and trimmed using Geneious R8 software [22]. Allele profiles and sequence type (ST) identity for each isolate were determined by entry of sequences and allelic profiles, respectively into the MLST online database. Clonal complexes (CC) were assigned using the eBURST algorithm (eBURST V3).

### Staphylococcus chromosome cassette typing and Panton-Valentine leukocidin detection

PCR-based genotyping of the chromosomal cassette recombinase (*ccr*) and *mec* complexes comprising the SCC*mec* was conducted using multiplex PCR [23]. Amplicons were generated using Q5 High Fidelity 2X Master Mix (New England BioLabs) and corresponding primers [23]. Reference strains USA100 (SCC*mec* type II), USA800 (SCC*mec* type IV) and USA300 (SCC*mec* type IV) were provided by the Network of Antimicrobial Resistance in Staphylococcus aureus (NARSA) and served as positive controls. Presence or absence of the Panton-Valentine Leukocidin (PVL) toxin gene was determined by direct PCR amplification of the *LukS-PV-lukF-PV* genes using GoTaq Green Master Mix (Promega) and previously described primers [24].

## Results

In this cross-sectional study, we evaluated the molecular epidemiology and antimicrobial resistance profile of 262 *S*. *aureus* isolates recovered between January and December, 2014 from patients seen at the Massachusetts Eye and Ear (Table 1). The isolates were routinely recovered from sinus (n = 139), ear (n = 67) and eye (n = 56) infections. This population comprised 78.2% MSSA (n = 205) and 21.7% MRSA (n = 57) isolates. The proportion of MRSA was not random across distinct infection categories. Otitis cases were significantly more resistant to methicillin (*P* = 0.01) and there was an apparent MRSA enrichment among isolates from orbital cellulitis (41%, P = 0.07) as opposed to isolates from keratitis (20%, P = 1.00) and conjunctivitis (23%, P = 1.00). The overall rates of resistance to clinically relevant antibiotics, population structure and enrichment of resistance phenotypes across major *S*. *aureus* lineages causing ocular and otolaryngology infections are presented.

### Resistance rates to clinically relevant antibiotics

**Fig 1:**
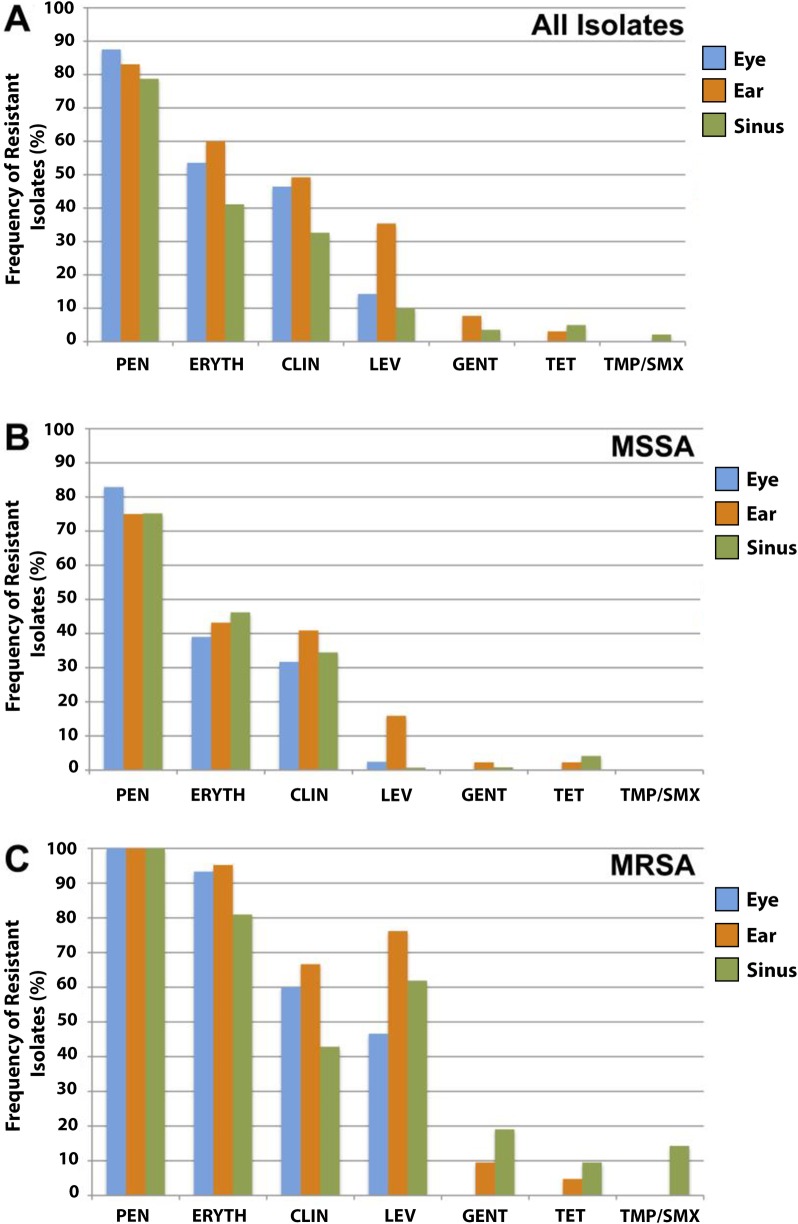
(A) Antimicrobial resistance screening of all *S*. *aureus* isolates. Frequency of individual antimicrobial resistances in MSSA (B) and MRSA (C) isolates against penicillin (PEN), erythromycin (ERYTH), clindamycin (CLIN) levofloxacin (LEV), gentamicin (GENT), tetracycline (TET), and trimethoprim/sulfamethoxazole (TMP/SMX).

To determine the rates of antibiotic resistance and the drugs that would predict better treatment outcomes of ocular and otolaryngology *S*. *aureus* infections based on *in vitro* susceptibility profiles, we tested the sensitivities of all isolates against a panel of commonly used antibiotics. Despite the ordinary community origins of our collection, we found moderate to high rates of resistance to clinically important antibiotics that are often used for treatment of infections affecting the eye and upper respiratory tract, such as penicillin (≥78%), erythromycin (≥41%) and clindamycin (≥32%). These resistances occurred at approximately equal rates among ocular, ear, and sinus isolates (Fig 1). Resistance rates to levofloxacin, part of an antibiotic class commonly used to treat otitis, conjunctivitis and keratitis, was markedly higher among ear isolates (35.4%) compared to other sites (14.3% for eye and 9.9% for sinus isolates). The resistance rates were overall affected by the methicillin resistance status, with MRSA isolates being consistently more resistant in comparison to MSSA isolates. The antibiotics mostly affected by the MRSA status were erythromycin, levofloxacin and clindamycin (Fig 1C).

Although MSSA resistance rates were lower compared to MRSA, the MSSA population was nonetheless frequently resistant to clinically relevant antibiotics. More than 70% of MSSA isolates were resistant to penicillin, and from 31% to 46% were resistant to erythromycin and clindamycin (Fig 1B). These data demonstrate that even among MSSA, antibiotics frequently used in our setting would cover only a fraction of patients infected by *S*. *aureus*. Resistance to levofloxacin was rare among ocular and sinus MSSA isolates (2.4% and 0.7%), but 16% of ear MSSA isolates were resistant to this antibiotic (Fig 1B).

Gentamicin, tetracycline and trimethoprim/sulfamethoxazole (TMP/SMX) demonstrated good *in vitro* coverage against both MSSA and MRSA isolates. None of the eye isolates were resistant to these antibiotics, and only a small fraction of resistant isolates were identified from ear (<10%) and sinus (<20%) infections. All MSSA and MRSA isolates were susceptible to the last-resort antibiotics linezolid, vancomycin and daptomycin.

### Population structure

To understand the population structure of *S*. *aureus* causing ocular and otolaryngology infections, our collection was typed using MLST. As determined by eBURST analysis, the population was largely comprised of 3 major clonal complexes, including CC5 (29.2%), CC30 (17.5%) and CC8 (14.8%) (Table 2). Stratified analysis demonstrated that these CCs were consistently the most prevalent across distinct body sites. Lineages isolated from ocular infections were mainly grouped in the CC5 (23.2%) and CC8 (17.8%), while ear and sinuses infections were dominated by CC5 (32.8% and 22.3%, respectively) and CC30 strains (19.4% for both sites) followed by CC8 (16.4% and 13.2%, respectively). These are the major and expanding CCs found in the USA, and contain successful community- and hospital-associated epidemic clones [25–27]. The remaining isolates were grouped in 19 different minor CCs, including some of the most common CCs found among colonizing or infecting *S*. *aureus* isolates from humans (e.g., CC45, CC15, CC72, CC1, CC59, CC398, CC25 and CC12) [25, 26].

**Table 2:** Distribution of major and minor CCs, proportion of MRSA and sites of isolation among 262 ocular and otolaryngology *S*. *aureus* isolates.

Clonal Complex	No. of isolates (% of total)	Proportion of MRSA (% of CC)	No. (% of total from site) of Isolates from:		
			Eye (n = 56)	Ear (n = 67)	Sinus (n = 139)
CC5	66 (25.2)	32 (48.5)	13 (23.2)	22 (32.8)	31 (22.3)
CC30	46 (17.5)	-	6 (10.7)	13 (19.4)	27 (19.4)
CC8	39 (14.8)	22 (55.0)	9 (16.0)	11 (16.4)	19 (13.6)
CC45	16 (6.1)	-	4 (7.1%)	3 (4.4%)	9 (6.5%)
CC15	15 (5.7)	-	5 (8.9%)	2 (3.0%)	8 (5.7%)
CC72	12 (4.6)	1 (8.3)	4 (7.1%)	1 (1.5%)	7 (5.0%)
CC1	11 (4.2)	-	4 (7.1%)	1 (1.5%)	6 (4.3%)
CC59	10 (3.8)	1 (10.0)	-	2 (3.0%)	8 (5.7%)
CC97	10 (3.8)		-	3 (4.4%)	7 (5.0%)
CC398	8 (3.0)	-	4 (7.1%)	2 (3.0%)	2 (1.4%)
CC25	8 (3.0)	-	3 (5.3%)	-	5 (3.6%)
CC188	6 (2.3)	-	1 (1.8%)	1 (1.5%)	4 (2.8%)
CC7	4 (1.5)	-	2 (3.5%)	-	2 (1.4%)
CC12	3 (1.1)	-	-	1 (1.5%)	2 (1.4%)
Others^1^	8 (3.0)	1	1 (1.8%)	5 (7.4%)	1 (0.7%)

^1^Sporadic STs. One isolate was found for each of the following: ST22, ST101, ST109, ST121, ST133, ST239, ST672, ST3089 (MRSA)

The occurrence of methicillin resistance was enriched among certain lineages, with the vast majority of MRSA isolates grouped in only two of the major CCs, namely CC5 (48.5%) and CC8 (55.0%) (Table 2). Together, these two CCs comprised almost half of the studied *S*. *aureus* population (40%). In contrast, only a small proportion of CC59 (10%) and CC72 (8.3%) strains were MRSA. A small number of isolates (3%) belonged to new STs, and one of these (ST3089, CC130) was resistant to methicillin. There was no reported resistance to methicillin among all remaining CCs found in our collection.

### MSSA population is dominated by five distinct lineages with varying rates of antibiotic resistance

**Fig 2:**
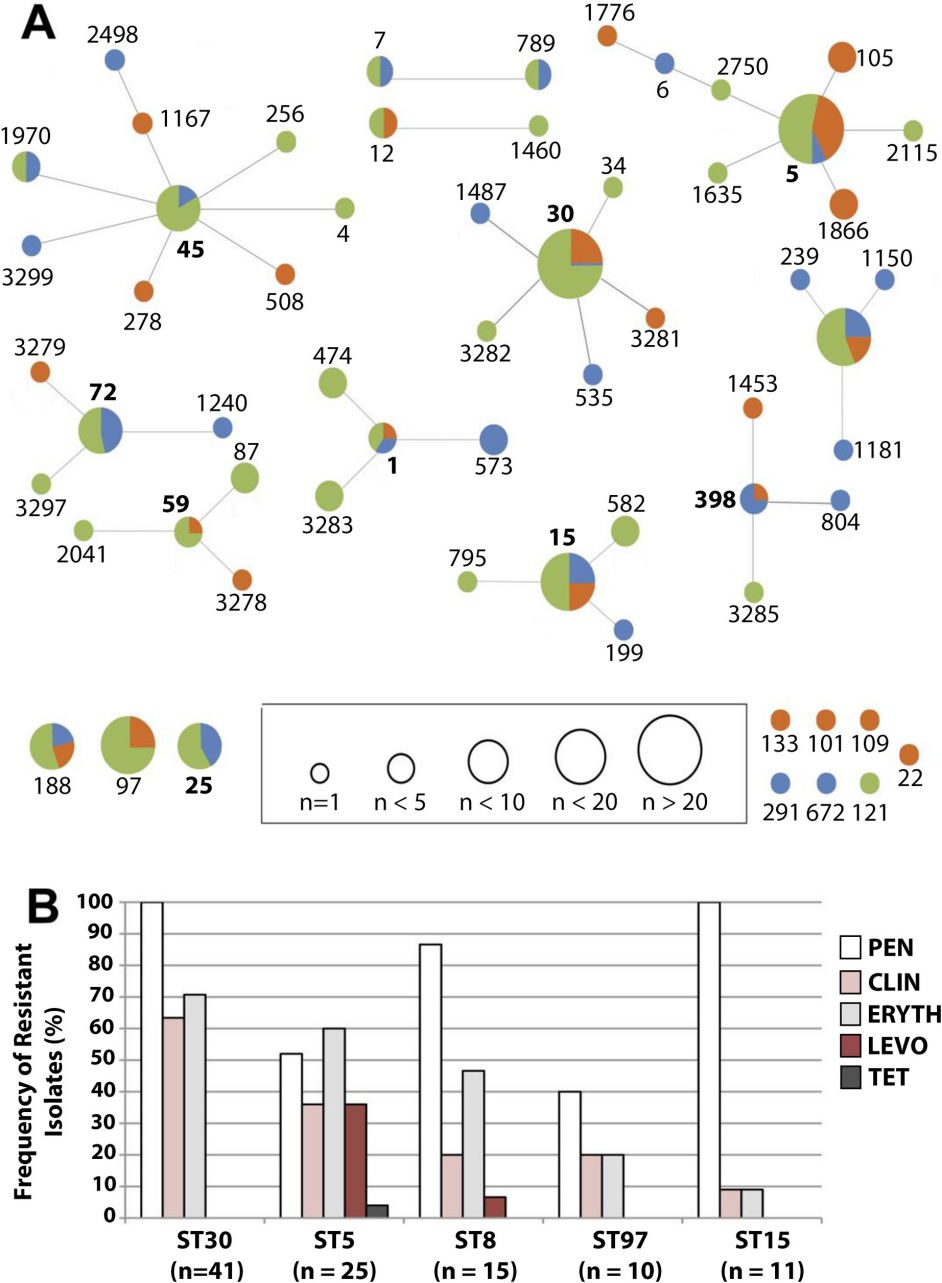
(A) eBURST population analysis of MLST allelic profiles of 205 MSSA isolates, organized by site of isolation. Circle sizes are reflective of ST frequency, and are colored based on the percentage of isolates collected from the eye (blue), ear (orange), or sinuses (green). Bolded numbers are indicative of the founder ST of a clonal complex. (B) Frequency of isolates resistant to penicillin (PEN), clindamycin (CLIN), erythromycin (ERYTH), levofloxacin (LEV), and tetracycline (TET) antimicrobials, sorted by prominent ST clonal lineages.

Prior efforts to understand the population structure of *S*. *aureus*, the major CCs in different settings and geographic locations, and the characteristics of the expanding epidemic clones have been largely focused in the subset of MRSA strains. Although MRSA is a major public health concern, MSSA strains are also able to cause drug-resistant and sometimes life-threatening infections [28, 29]. In addition, MSSA strains can acquire the SCC*mec* cassette, and some circulating genetic backgrounds may be poised to expand and become successful MRSA clones upon acquisition of this mobile element [27]. Thus we were motivated to understand the population structure and associated antibiotic resistances of MSSA isolates circulating in our unique ocular and otolaryngology population. As expected, the MSSA population was much more diverse in comparison to the MRSA population. As revealed by eBURST analysis, 195 of the 205 MSSA isolates belong to 63 different known STs associated with 21 CCs (Fig 2A). This population was, however, dominated by five major lineages, namely ST30 (n = 41), ST5 (n = 25) ST8 (n = 15), ST15 (n = 11), and ST97 (n = 10). While strains belonging to ST30, ST5, and ST8 are often found associated with humans, ST15 strains are also commonly associated with carriage and infection in humans [25], and ST97 is primarily a livestock-associated lineage that has been occasionally isolated from humans [30]. The distribution of these clones across distinct body sites appears to be random, with the exception of ST30 and ST97, which were enriched among sinus and ear isolates, and ST45, which was enriched among sinus and ocular isolates.

Resistance to penicillin, erythromycin and clindamycin was common among the major MSSA lineages, with fluctuations in the rates noticeably impacted by the genetic background (Fig 2B). Isolates belonging to the most predominant lineage, ST30, were uniformly resistant to penicillin and also had strikingly high rates of resistance to erythromycin (71%) and clindamycin (63%). Resistance to these 3 antibiotics (penicillin+clindamycin+erythromycin) was commonly found to co-occur (63.4%) in ST30 strains. ST5 and ST8 MSSA strains were also commonly resistant to penicillin, clindamycin and erythromycin, with slightly lower rates compared to ST30, and were the only major MSSA lineages displaying resistance to levofloxacin (36% and 7%, respectively). MSSA isolates belonging to the ST5 lineage, the genetic background of common successful hospital-associated MRSA clones, were the only to present resistance to 5 different antibiotic classes.

### MRSA strains belong to major community- and hospital-associated epidemic clones

**Fig 3:**
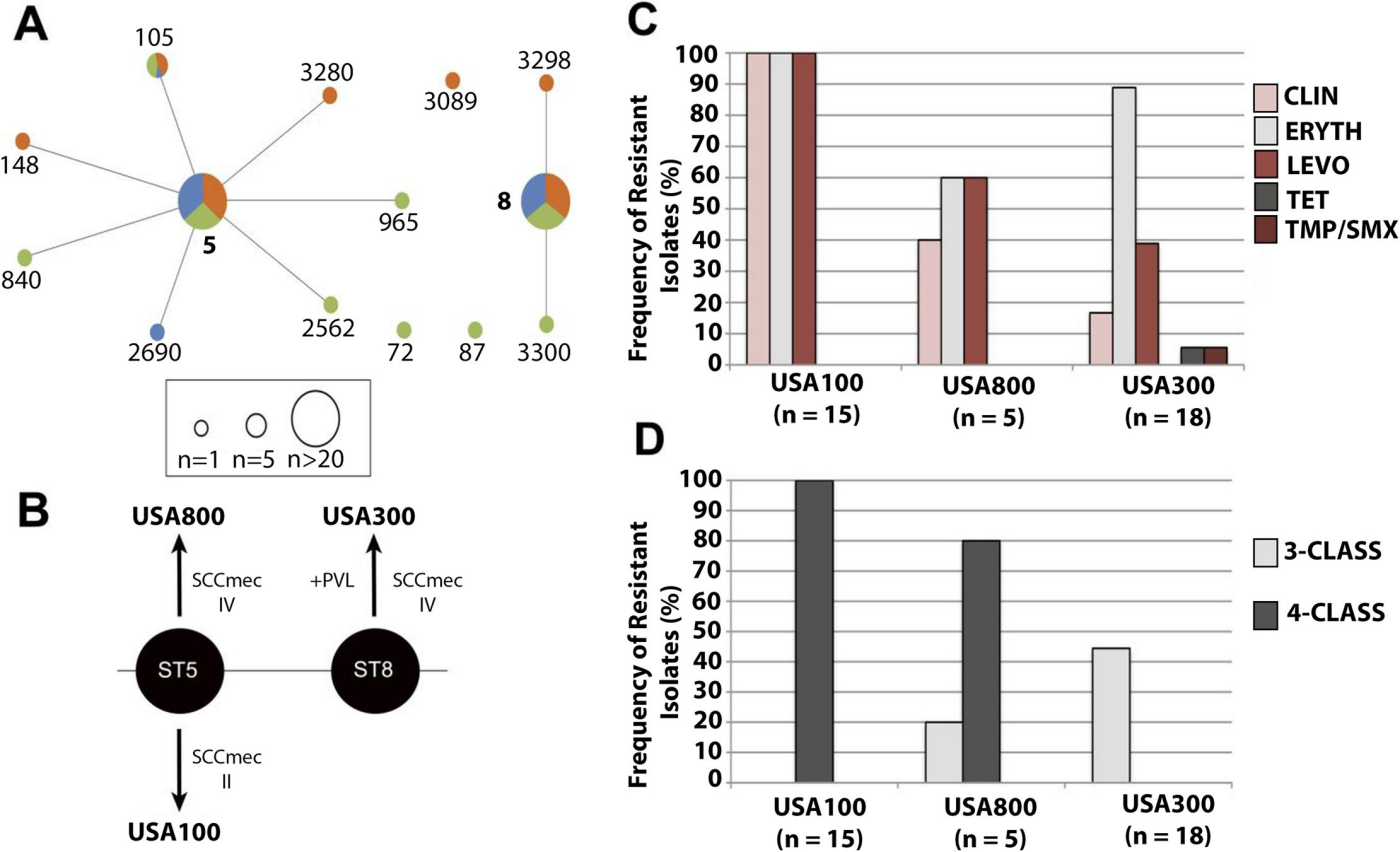
(A) eBURST population analysis of MLST allelic profiles of 57 MRSA isolates, organized by site of isolation. Circle sizes are reflective of ST frequency, and are colored based on the percentage of isolates collected from the eye (blue), ear (orange), or sinuses (green). Bolded numbers are indicative of the founder ST of a clonal complex. (B) Defining characteristics that differentiate the MRSA clonal lineages USA100, USA300, and USA800. (C) Antimicrobial resistance frequency to clindamycin (CLIN), erythromycin (ERYTH), levofloxacin (LEV), gentamicin (GENT), tetracycline (TET), and trimethoprim/sulfamethoxazole (TMP/SMX) among prominent clonal lineages and associated CCs. (D) Frequency of multidrug-resistant phenotypes within prominent clonal lineages and associated CCs.

Historically, MRSA infections in the US have been caused by two main clones that are predominant either in the hospital environment (clone USA100, NY/Japan), or in the community (clone USA300) [26]. By associating SCC*mec* types with MLST, we sought to determine whether these epidemiological findings would hold true for MRSA isolates from ocular and otolaryngology infections. As opposed to the MSSA population structure, MRSA isolates formed a less genetically diverse group. Only 14 different STs, grouped into 5 CCs, were found among MRSA isolates. CC5 (56.1%) and CC8 (38.6%) dominated the population (Fig 3A and Table 3). Most of the CC5 strains carried a SCC*mec* type II, which includes isolates with the characteristics of the USA100 clone, also known as NY/Japan (ST5-SCCmec-II), the main hospital-associated MRSA in the USA. All the CC8 strains were SCC*mec* type IV and most of them (86%) were positive for the PVL toxin, which are common features of the community-acquired MRSA clone USA300 (ST8-SCCmecIV, PVL+). These two MRSA types were equally prevalent in the population (38.6%), and represented the most common lineages (Fig 3B). CC5 strains harboring a SCC*mec* type IV, typical for the USA800 clone (also called the Pediatric Clone), comprised 15.5% of the population (Fig 3B). A single CC5 strain carried a SCC*mec* type V. The remaining isolates (n = 3) harbored a type II SCC*mec* and belonged to 3 different clonal complexes (Table 3).

**Table 3:** Molecular typing and presence of Panton-Valentine leukocidin (PVL) toxin among 57 ocular and otolaryngology MRSA isolates.

Clonal Complex (No.)	MRSA type	No. (% of total)	PVL (%)	No. of isolates from:		
				Eye	Ear	Sinus
CC5 (32)	CC5-SCC*mec*-II	22 (38.6%)	-	7	9	6
	CC5-SCC*mec*-IV	09 (15.8%)	-	2	3	4
	CC5-SCC*mec*-V	01 (1.7%)	-	-	-	1
CC8 (22)	CC8-SCC*mec*-IV	22 (38.6%)	19 (86.3%)	6	8	8
CC130 (1)	ST3089-SCC*mec*-II	1 (1.7%)	-	-	-	1
CC72 (1)	ST72-SCC*mec*-II	1 (1.7%)	-			1
CC59 (1)	ST87-SCC*mec*-II	1 (1.7%)	-	-	-	1

Resistance to clinically important antibiotics varied significantly between distinct MRSA subtypes (Fig 3C). In particular, strains resembling the USA100 clone were remarkably resistant to the antibiotics tested. In addition to beta-lactam resistance, 100% of the isolates were resistant to 3 additional classes of antibiotics, including clindamycin, erythromycin and levofloxacin. USA800-related isolates, the second major CC5 MRSA type in our collection, were also frequently resistant to clindamycin (40%), erythromycin (60%), and levofloxacin (60%). Unlike the CC5 isolates, USA300 strains exhibited lower multidrug-resistance rates (Fig 3D). The erythromycin resistance rate (89%) was the highest for USA300 isolates, followed by levofloxacin (39%). Clindamycin demonstrated a significantly better coverage of USA300 isolates (only 17% resistant) compared to the CC5 strains. Resistance to tetracycline and TMP/SMX was not observed among CC5 strains, and was sporadic for USA300 strains (6%).

## Discussion

Active surveillance and analysis of population structure and dynamics are important elements for the control of successful bacterial clones, especially for organisms for which the epidemiology can rapidly evolve, like *S*. *aureus* [31, 32]. Since the genetic landscape of *S*. *aureus* associated with common infections of the eye, ear, and sinus is not well defined, we sought to generate a snapshot of the population structure from these infection sites, as well as evaluate the related antimicrobial resistance profiles. We found that the *S*. *aureus* population from head and neck infections is composed of genetic lineages of both community and hospital origin, and that many MRSA and importantly MSSA strains are resistant to clinically important antibiotics.

The unique population of ocular and otolaryngology *S*. *aureus* isolates that we studied was dominated by major clonal complexes known to be associated with human colonization and infection (CC5, CC30 and CC8), in both community and hospital settings [25]. This suggests that these lineages are non-specific in their tissue tropism, and are able to dominate the bacterial population in a variety of distinct anatomical niches. In addition, strains from these genetic backgrounds have evolved to acquire and maintain antimicrobial resistance genes, which may be why they are among the most common causes of epidemics of antibiotic-resistant *S*. *aureus* infection [25, 27, 33]. Corroborating these findings, in our study CC5 and CC8 strains together made up 95% of the MRSA population, and were frequently co-resistant to additional antibiotic classes. CC30 isolates were not methicillin-resistant, but were however highly resistant to other clinically relevant antibiotics such as penicillin, clindamycin and erythromycin.

Although the *S*. *aureus* population we studied was dominated by a handful of clonal complexes, we observed a high diversity of individual STs (n = 64) grouped into several clonal complexes (n = 22). As expected, the MSSA population was much more diverse compared to the MRSA population [28, 34], and accounted for most of the population diversity. Out of 64 STs, only one was unique to the MRSA population, with the remaining 63 STs distributed across the MSSA population.

The major MSSA lineages found among our isolates have been commonly associated with human infection and colonization [25]. The single most frequent cluster, CC30, comprises the most common MSSA lineage in Europe [33, 35], and has given rise to important antibiotic-resistant epidemic clones, including the historic phage 80/81 strain, and more recently the EMRSA-16 and PVL-positive southwest Pacific (SWP) CA-MRSA clone [33]. A countrywide surveillance study in the USA has demonstrated that ST30 strains (*spa* typed as t012) were the third most common cause of MSSA bloodstream and skin and soft tissue infections between 2004 and 2010, and were widespread throughout the country [14]. Local epidemiology data from Illinois and Minnesota have also found CC30 as the first or second most frequent MSSA lineage isolated from clinically-relevant infections [28, 34]. Together, these and our data demonstrate the widespread dissemination and steady prevalence of CC30 in the population regardless of the site of isolation. Although none of the CC30 strains in our study were resistant to methicillin, their historic involvement as progenitors of successful antibiotic-resistant epidemic clones [33], and the high level of resistance to other clinically relevant antibiotics that we observed, underscore the importance of understanding the population structure of MSSA and its most predominant clones.

The MRSA population structure in our setting was notable for having much lower diversity compared to the MSSA population, and was represented by a handful of successful clones that display the molecular characteristics of the major USA MRSA clones. This population was dominated by CC5, carrying either a SCC*mec* type II (USA100) or type IV (USA800), and CC8 strains harboring a type IV SCC*mec* and frequently positive for the PVL toxin (USA300). The ocular and otolaryngology infections sampled in our study develop mainly in patients within the community setting, thus it was not surprising that we found strains related to the USA300 clone, the single most common CA-MRSA lineage in the USA, among the major MRSA lineages in our population [25]. Conversely, the CC5 strains in our study were related to USA100 and USA800, which have been found to be leading causes of antibiotic-resistant hospital-associated MRSA infections in the USA [36]. These results demonstrate that a heterogeneous population of lineages with both community and hospital origins composes the reservoir of primarily community-associated ocular and otolaryngology infections that we sampled. The USA100 clone, and less frequently USA800, have been reported among MRSA recovered from healthy carriers in the community and also from residents of nursing homes [37, 38], a finding that is further supported by the involvement of these lineages in community-associated skin and soft tissue infections as well as pneumonias in the USA [39]. Taken together with our results, it seems that MRSA lineages of hospital origin are widespread in the community, which may serve as an important reservoir for community-acquired infections affecting the eyes, ears, and sinuses.

Despite the community origins of the infections we characterized, we found numerous multidrug-resistant lineages that would impact the coverage of the most common antibiotics used for treatment of ocular and otolaryngology infections. Current antibiotic regimens for the majority of eye, ear, and sinus infections include various generations of fluoroquinolone, macrolide, lincosamide, and beta-lactam antibiotics [9, 40–43]. Amoxicillin, an aminopenicillin with a spectrum of activity similar to that of Penicillin G, is the drug of choice for treatment of sinusitis and acute otitis media, and is sometimes combined with the beta-lactamase inhibitor clavulanate [40, 42]. The major MSSA lineages in our study displayed high rates of resistance to penicillin, with some (ST30 and ST15) demonstrating total resistance. Although amoxicillin may be effective in the treatment of sinusitis and acute otitis media caused by other common bacterial pathogens like *S*. *pneumoniae*, *H*. *influenzae*, and *M*. *catarrhalis*, it would nonetheless be, in the vast majority of cases, completely ineffective against sinus and ear infections caused by *S*. *aureus*. Amoxicillin coupled with a beta-lactamase inhibitor would be a more appropriate option to improve coverage for sinus and ear infections caused by MSSA, and this combination has the added advantage of improved efficacy against other common respiratory pathogens that may produce beta-lactamases, such as *H*. *influenzae* and *M*. *catarrhalis*. Fluoroquinolones are largely used for the treatment of bacterial conjunctivitis, keratitis, and chronic otitis, and can also be used as an alternative antibiotic for sinusitis [9, 41–43]. Levofloxacin displayed good *in vitro* coverage against our MSSA population, but would have limited efficacy against MRSA strains, especially USA100 strains, which we found were completely resistant to this fluoroquinolone agent. Macrolides and clindamycin are considered alternative options for the management of sinusitis and otitis media [40, 42, 44]; these antibiotics would have very limited efficacy against MRSA infections caused by the USA100, USA800 and USA300 lineages, as well as many ST30, ST5 and ST8 MSSA cases.

Although the rates of resistance to methicillin in our population were lower than that seen among hospitalized patients [1], approximately one fourth of our *S*. *aureus* isolates (21%) were nonetheless resistant to this important antibiotic. As demonstrated above, we found that resistance to methicillin was often accompanied by higher rates of co-resistance to other antimicrobial classes. In particular, ocular and otolaryngology MRSA strains were noticeably more resistant to clindamycin, erythromycin and levofloxacin when compared to MSSA isolates. Our antimicrobial susceptibility testing suggests that tetracycline antibiotics, as well as trimethoprim/sulfamethoxazole (TMP/SMX), could be good options for empirical treatment of most MRSA ocular and otolaryngology infections. Prior studies of the efficacy of tetracycline and TMP/SMX against MRSA support our observations, especially since these two drug classes are well suited for use in respiratory infections due to their polymicrobial activity in the respiratory tract [45]. Indeed, the Infectious Disease Society of America recommends the use of TMP/SMX and tetracycline for treatment of non-complicated MRSA infections [46]. Finally, we found no evidence of resistance to vancomycin, daptomycin, and linezolid among our isolates, which is encouraging for their continued use as last-resort options for treatment.

Overall our findings demonstrate that the *S*. *aureus* population causing eye, ear, and sinus infections is composed of a heterogeneous mix of lineages that have been widely found in both community and hospital settings. It seems that the traditional community-hospital boundaries may be blurred by constant selection and expansion of multiple lineages that can thrive in both settings. Due to variations in the association of antibiotic resistances and genetic background observed in our study, it is apparent that proper and consistent monitoring of these lineages should be implemented, to further expand our understating of the population dynamics in this setting and to help promote the implementation of effective control measurements for *S*. *aureus* infections of the eyes, ear and sinuses.

## Supporting information

S1 Table De-identified dataset. (XLS)
